# Recent Advancements in Computer & Software Technology

**DOI:** 10.1155/2014/609512

**Published:** 2014-06-05

**Authors:** K. K. Mishra, A. K. Misra, Peter Mueller, Gregorio Martinez Perez, Sanjiv K. Bhatia, Yong Wang

**Affiliations:** ^1^Department of Computer Science, MNNIT, Allahabad, India; ^2^IBM Zurich Research Laboratory, Saeumerstraße 4, 8803 Rueschlikon, Switzerland; ^3^University of Murcia, Murcia, Spain; ^4^University of Missouri-St. Louis, MO, USA; ^5^School of Information Science and Engineering, Central South University, Changsha, China

Over the last few decades, advancements in computer and software technologies have reached an impressive level. These technologies improve not only very common areas of our daily life, but also areas of education, health, production industries, and so on. Thus, recent advancements in computer and software technologies are the base for the society of tomorrow.

The feasibility of future developments strongly relies on the existence of key technologies and their deployments. Analyzing global trends in cloud computing including all its services reveals cornerstone fields, such as distributed parallel processing, advanced software engineering, image processing, and security solutions. These fields require different sets of resources like computing hardware, Internet, software and hardware tools, mobility technologies, storage, system management, and security technology.

The objective of this special issue is to present a collection of articles that cover recent research results and comprehensive reviews on relevant computer and software technologies. In particular, it aims to present highly technical papers describing the areas mentioned above: distributed parallel processing, advanced software engineering, image processing, and security solutions.

This special issue received an overwhelming response from the community. Due to the limited space, only 10 papers from the initial 109 manuscripts have been selected. These papers represent the most up-to-date research work covering topics such as software process improvement and software testing, vulnerability and video quality assessment, artificial immune systems, improvements in file systems, and component models for distributed simulation, amongst others. Due to the space limitation we will not reiterate the contents here. We hope the reader will find this special issue informative and stimulating.

We would like to thank all the contributors who have submitted their high quality papers.

*K. K. Mishra* *K. K. Mishra* *A. K. Misra* *A. K. Misra* *Peter Mueller* *Peter Mueller* *Gregorio Martinez Perez* *Gregorio Martinez Perez* *Sanjiv K. Bhatia* *Sanjiv K. Bhatia* *Yong Wang* *Yong Wang*

